# Urine Immunoglobin G Greater Than 2.45 mg/L Has a Correlation with the Onset and Progression of Diabetic Kidney Disease: A Retrospective Cohort Study

**DOI:** 10.3390/jpm13030452

**Published:** 2023-02-28

**Authors:** Cheng Meng, Jiujing Chen, Xiaoyue Sun, Shilin Guan, Hong Zhu, Yongzhang Qin, Jingyu Wang, Yongmei Li, Juhong Yang, Baocheng Chang

**Affiliations:** 1 NHC Key Laboratory of Hormones and Development, Tianjin Key Laboratory of Metabolic Diseases, Chu Hsien-I Memorial Hospital & Tianjin Institute of Endocrinology, Tianjin Medical University, Tianjin 300134, China; 2 Department of Epidemiology and Biostatistics, School of Public Health, Tianjin Medical University, Tianjin 300134, China

**Keywords:** immunoglobulin G, type 2 diabetes mellitus, 24-h urinary albumin excretion rate, estimated glomerular filtration rate

## Abstract

Aim: To further assess the correlation between urine immunoglobin G (IgG) greater than 2.45 mg/L and the onset and progression of diabetic kidney disease (DKD). Methods: One thousand and thirty-five patients with type 2 diabetes mellitus (T2DM) were divided into two groups based on the baseline levels of 24 h urinary albumin excretion (24 h UAE): one group with 24 h UAE < 30 mg/24 h and one with 24 h UAE ≥ 30 mg/24 h. The groups were subdivided using baseline levels of urine IgG (≤2.45 mg/L and >2.45 mg/L; hereafter, the Low and High groups, respectively). We used logistic regression to assess the risk of urine IgG and it exceeding 2.45 mg/L. Kaplan–Meier curves were used to compare the onset and progression time of DKD. The receiver operating characteristic curve was used to test the predictive value of urine IgG exceeding 2.45 mg/L. Results: Urine IgG was an independent risk factor for the onset and progression of DKD. The rate and risk of DKD onset and progression at the end of follow-up increased significantly in the High group. The onset and progression time of DKD was earlier in the High group. Urine IgG exceeding 2.45 mg/L has a certain predictive value for DKD onset. Conclusions: Urine IgG exceeding 2.45 mg/L has a correlation with the onset and progression of DKD, and it also has a certain predictive value for DKD onset.

## 1. Introduction

DKD has become the leading cause of chronic kidney disease (CKD) with the increasing incidence of diabetes [1]. Microalbuminuria is a well-known predictor of DKD worsening but not of DKD, because DKD is often present at early stages with elevated glomerular filtration without microalbuminuria [2]. It is often difficult to reverse DKD after structural changes to the kidney occur. Consequently, there is an urgent need for biomarkers that can accurately diagnose early-stage DKD.

Lots of biomarkers related to DKD have been identified [3,4]. However, since most of them have not been clinically verified, their applicability in clinical settings is limited [5]. Recently, the association between IgG and kidney disease has attracted widespread attention. Research on IgG in nephropathy has shown that an increased IgG excretion rate appears to signify a decrease in the estimated glomerular filtration rate (eGFR) and an increase in segmental glomerulosclerosis and may be a sign of disease progression [6]. Increased urinary IgG reflects severe glomerular damage accompanied by greater proteinuria [7]. Studies have shown that urinary IgG can increase before the appearance of microalbuminuria [8].

In addition, a 5-year follow-up research of type 2 diabetics with normal albuminuria at baseline found that an increase in IgG had a good predictive value for microalbuminuria [9]. Additionally, our preliminary cross-sectional study found that urine IgG was a reliable predictor of DKD, with a cut-off point of 2.45 mg/L (the sensitivity was 80%, and the specificity was 70.2%) [5]. However, the correlation between urine IgG exceeding 2.45 mg/L and the onset and progression of DKD required further verification. As a validation cohort, this research was to further evaluate the correlation between them.

## 2. Methods

### 2.1. Study Objects

The recruitment criteria were a T2DM diagnosis, 18 years or older, follow-up time greater than 24 months, 24 h UAE < 300 mg/24 h, and complete relevant laboratory indicators during hospitalisation. We used the diagnostic criteria and classification system adopted for diabetes mellitus by the World Health Organization in 1999 to identify diabetics [10]. eGFR was according to the 2012 clinical practise guidelines Kidney Disease: Improving Global Outcomes (KDIGO) and the Chronic Kidney Disease Epidemiology Collaboration formula [11,12]. Thus, 1190 patients admitted to Tianjin Medical University Chu Hsien-I Memorial Hospital for the treatments of diabetes from June 2014 to April 2021 were enrolled.

**Figure 1:**
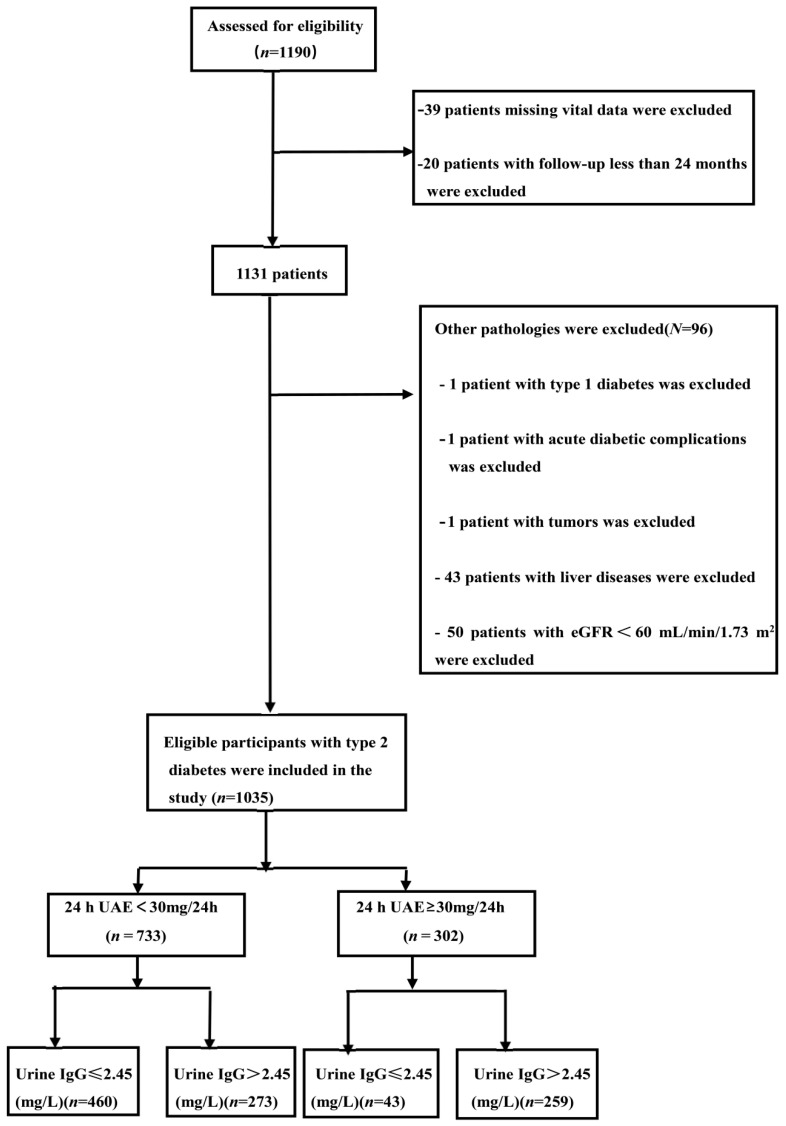
The flowchart of enrolment. 24 h UAE, 24-h urinary albumin excretion; DKD, diabetic kidney disease; eGFR, estimated glomerular filtration rate; IgG, immunoglobulin G.

Since kidney function may be affected by other kidney diseases, increased albuminuria, and hospitalisation opportunities, we eliminated patients with a history of primary nephrotic syndromes, chronic glomerulonephritis, lupus nephritis, urinary tract infection, acute kidney injury, urinary calculi, polycystic kidney disease, renal tubular injury, gout-associated nephropathy, hypertensive nephropathy, pyelonephritis, and interstitial nephropathy, such as gouty nephropathy. After eliminating 39 patients missing vital data, 1 type 1 diabetic participants, 1 patient with acute diabetic complications, 1 patient with tumours, 20 patients with follow-up less than 24 months, 50 patients affected by other kidney diseases with eGFR smaller than 60 mL/min/1.73 m^2^, and 43 patients with liver diseases, as a result, 1035 type 2 diabetics were incorporated into this study. The selection process is shown in Figure 1.

All participants analysed in this study provided written informed consent. The study followed the principles of the Declaration of Helsinki and was reviewed and approved by the Medical Ethics Committee of Tianjin Medical University Chu Hsien-I Memorial Hospital.

### 2.2. Definition of Onset and Progression of DKD

DKD is a chronic kidney disease peculiar to diabetes mellitus, containing albuminuria urine albumin to creatinine ratio (ACR) ≥ 30 mg/g, urinary albumin excretion rate (UAE) ≥ 30 mg/24 h or eGFR < 60 mL/min/1.73 m^2^ present for more than three months [13]. In this study, we excluded patients with baseline eGFR < 60 mL/min/1.73 m^2^ and/or 24 h UAE ≥ 300 mg/24 h.

#### 2.2.1. Definition of the Onset of DKD

Among patients with 24 h UAE < 30 mg/24 h at baseline, those with eGFR ≥ 60 mL/min/1.73 m^2^ and 24 h UAE < 30 mg/24 h at the end of follow-up were categorised as ‘no onset’, those with eGFR < 60 mL/min/1.73 m^2^ and 24 h UAE < 30 mg/24 h were categorised as ‘onset1’, those with eGFR ≥ 60 mL/min/1.73 m^2^ and 24 h UAE ≥ 30 mg/24 h were categorised as ‘onset2’, and those with eGFR < 60 mL/min/1.73 m^2^ and 24 h UAE ≥ 30 mg/24 h were categorised as ‘onset3’.

#### 2.2.2. Definition of the Progression of DKD

Among patients with baseline 24 h UAE between 30 and 300 mg/24 h, those with eGFR≥ 60 mL/min/1.73 m^2^ and 24 h UAE < 300 mg/24 h at the end of follow-up were categorised as ‘nonprogress’, those with eGFR < 60 mL/min/1.73 m^2^ and 24 h UAE < 300 mg/24 h were categorised as ‘progress1’, those with eGFR ≥ 60 mL/min/1.73 m^2^ and 24 h UAE ≥ 300 mg/24 h were categorised as ‘progress2’, and those with eGFR < 60 mL/min/1.73 m^2^ and 24 h UAE ≥ 300 mg/24 h were categorised as ‘progress3’.

### 2.3. Data Collection

We collected data on sex, age, BMI, course of diabetes, blood pressure, and other demographic and clinical information by interviewing patients and validating their responses against their medical records. We carefully recorded medication and smoking history. All hospitalised patients had undergone ophthalmic professional examinations to diagnose diabetic retinopathy. All blood samples were collected after 12 h of fasting. We used the AU5800 automatic biochemical analyser to analyse serum uric acid (SUA), serum creatinine (Scr), and blood lipids. We used the HLC-723G8 HbA1c analyser to measure haemoglobin A1c (HbA1c). All subjects provided 10 mL samples of clean midstream morning urine. The urine specimens were evaluated for β2-microglobulin (β2MG), IgG, and retinol-binding protein (RBP) using a Cobas8000 modular analyser. We collected 24 h urine for 2 successive days and used the average value in order to test the 24 h UAE level.

We tested all specimens in the Laboratory of Tianjin Medical University Chu Hsien-I Memorial Hospital. The ranges that the kit manufacturer gives for urine biomarkers are as follows: IgG, 0.0–17.5 mg/L; RBP, 0.0–0.7 mg/L; β2-MG, 0.0–0.3 mg/L.

### 2.4. Statistical Analysis

SPSS statistical software commercial version 22.0 (IBM, Chicago, IL, USA) was used to analyse the data. Sample sizes were estimated according to the factors included in the model. We used analysis of variance (ANOVA) and logistic regression to analyse differences between test groups. The onset and progression of DKD were regarded as the dependent variables. We used the log rank method and Kaplan–Meier curves to compare differences in the onset and progression time of DKD between the urine IgG ≤ 2.45 mg/L group and the urine IgG > 2.45 mg/L group. The receiver operating characteristic curve was used to test the predictive value of urine IgG > 2.45 mg/L for the onset and progression of DKD. All statistical tests were two-tailed, and *p*-values less than 0.05 were considered significant.

Descriptive statistics are shown as means and standard deviation (SD) or medians with interquartile ranges (IQR) of continuous variables and percentages for categorical variables. Quantitative data for normal and non-normal distribution were expressed as the mean ± standard deviation and median (first quartile, third quartile). We used independent-sample *t*-tests and nonparametric tests to analyse differences between groups for data with normal and non-normal distributions. 

## 3. Results

### 3.1. Baseline Features of Samples

Among patients with baseline 24 h UAE < 30 mg/24 h, we found significant differences between the High group (urine IgG > 2.45 mg/L) and the Low group (urine IgG ≤ 2.45 mg/L) in BMI, SBP, GGT, TG, HDL, urine β2-MG, urine IgG, urine RBP, and 24 h UAE. It is noteworthy that 24 h UAE was obviously higher in the High group (urine IgG > 2.45 mg/L) than in the Low group (urine IgG ≤ 2.45 mg/L) (*p* < 0.001) (Table 1).

Among patients with baseline 24 h UAE ≥ 30 mg/24 h, significant differences between groups were found in eGFR, sex, urine IgG, urine RBP, and 24 h UAE. It is noteworthy that 24 h UAE was obviously higher in the High group (urine IgG > 2.45 mg/L) than in the Low group (urine IgG ≤ 2.45 mg/L) (*p* < 0.001) (Table 1).

### 3.2. Rates of the Onset and Progression of DKD between Groups

**Figure 2:**
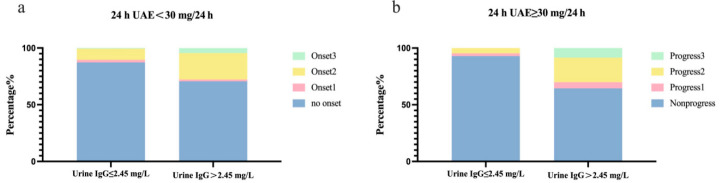
Rates of onset and progression of DKD. (**a**) Rates of onset of DKD. (**b**) Rates of progression of DKD. Among patients with baseline 24 h UAE < 30 mg/24 h, those with eGFR ≥ 60 mL/min/1.73 m^2^ and 24 h UAE < 30 mg/24 h at the end of follow-up were categorised as ‘no onset’, those with eGFR < 60 mL/min/1.73 m^2^ and 24 h UAE < 30 mg/24 h were categorised as ‘onset1’, those with eGFR ≥ 60 mL/min/1.73 m^2^ and 24 h UAE ≥ 30 mg/24 h were categorised as ‘onset2’, and those with eGFR < 60 mL/min/1.73 m^2^ and 24 h UAE ≥ 30 mg/24 h were categorised as ‘onset3’. Among patients with baseline 24 h UAE between 30 and 300 mg/24 h, those with eGFR ≥ 60 mL/min/1.73 m^2^ and 24 h UAE < 300 mg/24 h at the end of follow-up were categorised as ‘nonprogress’, those with eGFR < 60 mL/min/1.73 m^2^ and 24 h UAE < 300 mg/24 h were categorised as ‘progress1’, those with eGFR ≥ 60 mL/min/1.73 m^2^ and 24 h UAE ≥ 300 mg/24 h were categorised as ‘progress2’, and those with eGFR < 60 mL/min/1.73 m^2^ and 24 h UAE ≥ 300 mg/24 h were categorised as ‘progress3.’ 24 h UAE, 24 h urinary albumin excretion; DKD, diabetic kidney disease; IgG, immunoglobulin G.

As the results (Supplementary Table S1) revealed statistical significance between urine IgG and the onset and progression of DKD, we further evaluated the correlation between the cut-off point 2.45 mg/L (found in our previous study [5]) and DKD. The rate and risk of DKD onset and progression at the end of follow-up increased significantly in the High group. Additionally, the rates of urine IgG > 2.45 mg/L were significantly higher in onset2 and onset3 than in no onset (Table 2 and Figure 2a), and it was significantly higher in progress2 than in no progress too (Table 2 and Figure 2b).

### 3.3. The Relationship between Urine IgG Greater Than 2.45 mg/L and the Onset and Progression of DKD

Among patients with 24 h UAE < 30 mg/24 h, univariate regression revealed that the High group (urine IgG > 2.45 mg/L) had a significantly increased risk for onset2 and onset3 at the end of follow-up compared to the Low group (urine IgG ≤ 2.45 mg/L) (OR = 2.959, 95% CI: 1.949–4.505; OR = 8.333, 95% CI: 2.326–30.303, respectively). Furthermore, multiple regression revealed the same effects (OR = 2.617, 95% CI: 1.623–4.219; OR = 14.706, 95% CI: 2.188–100.000, respectively) (Table 3). 

Among patients with 24 h UAE ≥ 30 mg/24 h, univariate regression revealed that the High group (urine IgG > 2.45 mg/L) had a significantly higher risk for progress2 at the end of follow-up compared to the urine IgG ≤ 2.45 mg/L group (OR = 6.711, 95% CI: 1.570–28.571). Multiple regression revealed the same effects (OR = 7.353, 95% CI: 1.475–37.037). (Table 3).

### 3.4. Kaplan–Meier Curves for DKD Onset and Progression

**Figure 3:**
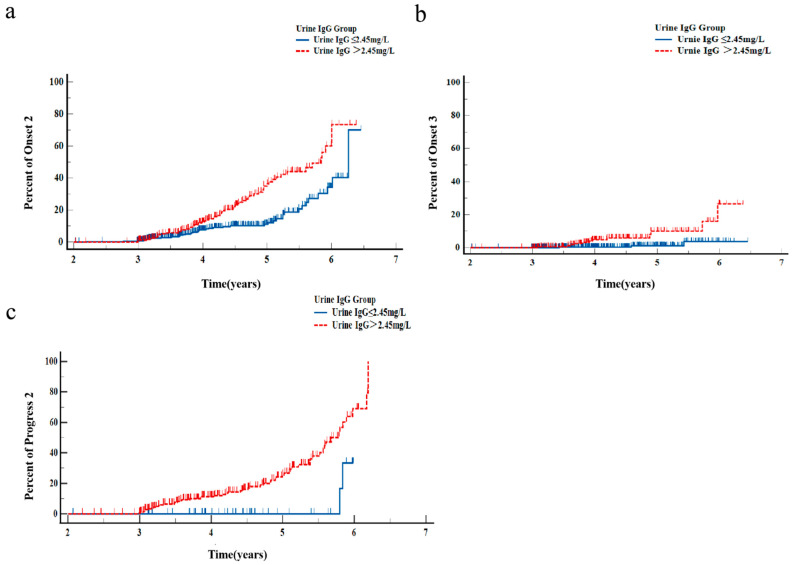
Kaplan–Meier curves for DKD onset and progression. (**a**) Using onset2 as a dependent variable, urine IgG ≤ 2.45 mg/L and urine IgG > 2.45 mg/L, the estimated median onset times, were 6.261 years and 5.836 years, respectively (χ^2^ = 19.960, *p* < 0.001). (**b**) Using onset3 as a dependent variable, urine IgG ≤ 2.45mg/L and urine IgG > 2.45 mg/L, the estimated median onset times, were 6.404 years and 6.087 years, respectively (χ^2^ = 12.717, *p* < 0.001). (**c**) Using progress2 as a dependent variable, urine IgG ≤ 2.45 mg/L and urine IgG > 2.45 mg/L, the estimated median onset times, were 5.929 years and 5.394 years, respectively (χ^2^ = 8.618, *p* = 0.0033). Among patients with baseline 24 h UAE < 30 mg/24 h, those with eGFR ≥ 60 mL/min/1.73 m^2^ and 24 h UAE < 30 mg/24 h at the end of follow-up were categorised as ‘no onset’, those with eGFR ≥ 60 mL/min/1.73 m^2^ and 24 h UAE ≥ 30 mg/24 h were categorised as ‘onset2’, and those with eGFR < 60 mL/min/1.73 m^2^ and 24 h UAE ≥ 30 mg/24 h were categorised as ‘onset3’. Among patients with baseline 24 h UAE between 30 and 300 mg/24 h, those with eGFR ≥ 60 mL/min/1.73 m^2^ and 24 h UAE ≥ 300 mg/24 h at the end of follow-up were categorised as ‘progress2’. 24 h UAE, 24-h urinary albumin excretion; DKD, diabetic kidney disease; IgG, immunoglobulin G.

Since urine IgG > 2.45 mg/L had a significant correlation with onset2, onset3, and progress2, we further explored the onset and progression time between them. Among patients with 24 h UAE *<* 30 mg/24 h, the onset (onset2 and 3) time in the High group (urine IgG > 2.45 mg/L) was significantly earlier than in the Low group (urine IgG ≤ 2.45 mg/L) (χ^2^ = 19.960, *p* < 0.001; χ^2^ = 12.717, *p* < 0.001, respectively) (Figure 3a,b). Among patients with 24 h UAE ≥ 30 mg/24 h, the progression (progress2) time in the High group (urine IgG > 2.45 mg/L) was also significantly earlier than in the Low group (urine IgG ≤ 2.45 mg/L) (χ^2^ = 8.618, *p =* 0.0033) (Figure 3c).

### 3.5. Receiver Operating Characteristic Curves for DKD Onset and Progression

**Figure 4:**
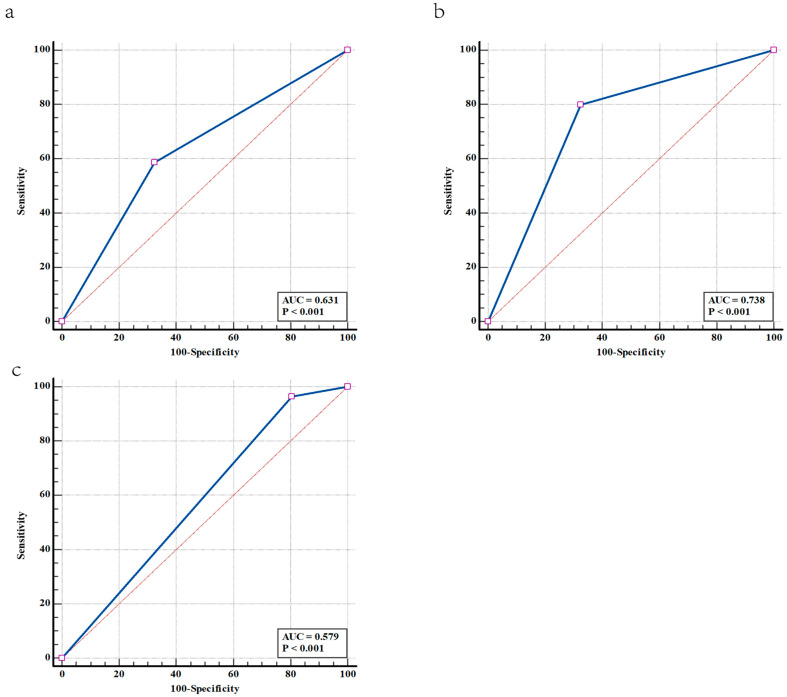
The value of urine IgG > 2.45 mg/L in predicting onset2, onset3 and progress2. (**a**) Receiver operating characteristic curve of urine IgG > 2.45 mg/L in predicting onset2. The AUC and its 95%CI were 0.631 (0.595–0.667). (**b**) Receiver operating characteristic curve of urine IgG > 2.45 mg/L in predicting onset3. The AUC and its 95%CI were 0.738 (0.701–0.772). (**c**) Receiver operating characteristic curve of urine IgG > 2.45 mg/L in predicting progress2. The AUC and its 95%CI were 0.579 (0.517–0.640). Among patients with baseline 24 h UAE < 30 mg/24 h, those with eGFR ≥ 60 mL/min/1.73 m^2^ and 24 h UAE < 30 mg/24 h at the end of follow-up were categorised as ‘no onset’, those with eGFR ≥ 60 mL/min/1.73 m^2^ and 24 h UAE ≥ 30 mg/24 h were categorised as ‘onset2’, and those with eGFR < 60 mL/min/1.73 m^2^ and 24 h UAE ≥ 30 mg/24 h were categorised as ‘onset3’. Among patients with baseline 24 h UAE between 30 and 300 mg/24 h, those with eGFR ≥ 60 mL/min/1.73 m^2^ and 24 h UAE ≥ 300 mg/24 h at the end of follow-up were categorised as ‘progress2’. 24 h UAE, 24-h urinary albumin excretion; DKD, diabetic kidney disease; IgG, immunoglobulin G.

Since urine IgG > 2.45 mg/L had a significant correlation with onset2, onset3, and progress2, we further examined the predictive value of urine IgG > 2.45 mg/L to them. The area under the curves (AUC) and 95% CI of onset2, onset3, and progress2 were 0.631(0.595– 0.667) (Figure 4a), 0.738 (0.701–0.772) (Figure 4b) and 0.579 (0.517–0.640) (Figure 4c), respectively. Thus, urine IgG > 2.45 mg/L had a certain predictive value for DKD onset, especially for onset3.

## 4. Discussion

In the present study, we found that urine IgG was an independent risk factor for the onset and progression of DKD. Urine IgG greater than 2.45 mg/L had a correlation with the onset and progression of DKD, and it also had a certain predictive value for DKD onset. In addition, the onset and progression time of DKD were earlier in the High group (urine IgG > 2.45 mg/L).

IgG, a marker of increased glomerular permeability, was significantly associated with urinary albumin. This indicates that the supposed greater glomerular permeability leads to increased excretion of IgG and albumin, the latter of which is typically excreted at lower levels [7]. Several studies have reported increases in IgG in normoalbuminuric diabetic patients [14,15,16].

In addition, five years follow-up research revealed that increased urine IgG excretion could predict microalbuminuria in patients with T2DM [9]. However, the cut-off point of urine IgG for predicting the onset and progression of DKD in type 2 diabetics was rarely explored. In our previous study, we found that urine IgG was an important predictor of DKD. The cut-off point after propensity score matching was 2.45 mg/L [5]. However, the correlation between urine IgG > 2.45 mg/L and the onset and progression of DKD needs to be further verified.

In the present validation cohort study, we found that urine IgG was an independent risk factor for the onset and progression of DKD. Additionally, the rates of onset and progression of DKD were obviously increased in people with urine IgG greater than 2.45 mg/L at baseline, which provided direct evidence of a potential correlation between urine IgG greater than 2.45 mg/L and the onset and progression of DKD.

Additionally, analysis of logistic regression revealed that the High group (urine IgG > 2.45 mg/L) was more likely to suffer the onset and progression of DKD at the end of follow-up than the Low group. This further validated the correlation of urine IgG levels greater than 2.45 mg/L with them. Early detection of urinary IgG appears somewhat effective for delaying the onset and progress of DKD. The receiver operating characteristic curve revealed that urine IgG greater than 2.45 mg/L had certain predictive value for the onset of DKD, especially for the simultaneous onset of abnormal 24 h UAE and eGFR and provided certain reference value for the early diagnosis of DKD. Thus, it may be an important clinical indicator. Our conclusion verified its predictive value to the onset of DKD, and further found that it also had significant correlation with the progression of DKD. It is worthy of further research to verify its predictive ability for the progression of DKD.

In the present study, urine IgG greater than 2.45 mg/L was not correlated with the reduced eGFR with stationary 24 h UAE, which was consistent with our previous findings. Reduced eGFR with stationary 24 h UAE may be related to reasons other than diabetic kidney disease, such as fluctuations in blood pressure.

Urinary IgG excretion was analysed as an indicator of pore size selective damage in the renal globules [17,18,19]. According to a previous study [20], the rate of urine IgG excretion may increase in conditions as follows: pore size selective damage of glomerulus and enhanced intraglomerular hydraulic pressure. Urine IgG may reflect variations in renal hemodynamics, with a greater sensitivity than microalbuminuria [9]. Given that hyperglycaemia increases the intraglomerular hydraulic pressure [21,22,23] in the early stages of diabetic nephropathy, parallel increases in urinary IgG probably reflect increased intraglomerular hydraulic pressure. Increased intraglomerular hydraulic pressure does not increase albuminuria [9]. Our results revealed that HbA1c was an independent predictor of microalbuminuria. Therefore, it was speculated that the hyperglycaemic environment caused by diabetes induces an increase in intraglomerular hydraulic pressure, leading to increased excretion of urine IgG prior to 24 h UAE. It suggests that, in a diabetic patient with normal kidney function, the changes in urinary IgG precedes microalbuminuria to reflect an abnormal renal function.

Our results also indicated that haemoglobin A1c and age were also associated with the onset and progression of DKD. Many trials have shown that the strict management of blood glucose (HbA1c 6.5–7.0%) can reduce the risk for DKD [24,25,26,27]. The earlier antihyperglycemic therapy starts, the better the prognostic benefits [28]. Jiang et al. reported that the risk for DKD increased by 17% with a 1% increase in the HbA1c levels [29]. In addition, increases in the urinary excretion of albumin and IgG induced by diabetes were more readily normalised by euglycemia [30,31]. Our results were consistent with previous research. This indicated that the onset and progression probability of DKD increased considerably with increases in glycosylated haemoglobin.

In previous studies, the elderly were generally considered to be an adverse factor for DKD development [32]. Russo et al. found that the prevalence of DKD was higher in people with type 2 diabetes who are over 65 years of age [33]. Age-dependent changes in DKD morbidity and risk factors may be associated with age related hormonal changes [34]. Our results revealed same effects, suggesting that age was a factor in DKD onset that cannot be ignored.

In conclusion, this retrospective study confirmed that urine IgG greater than 2.45 mg/L had a correlation with the onset and progression of DKD, especially with 24 h UAE, thus providing a new route to the diagnosis of early staged DKD. However, this study was finished in a single clinical centre, and the versatility of the sample was limited. Therefore, further multicentre studies are needed to assess the correlation.

Personalised part: At present, the early diagnosis of diabetic kidney disease is mainly dependent on microalbuminuria, and the renal function changes, such as increased glomerular filtration rate, have occurred before the onset of the symptom. Therefore, more accurate and personalised prevention and diagnosis for early diabetic kidney disease patients are urgently needed. The present study revealed that urine immunoglobin G exceeding 2.45 mg/L had a correlation with the onset and progression of diabetic kidney disease, and it also had a certain predictive value for the onset of it. Thus, we used the data of each individual to make the decision to provide a personalized reference interval for the prevention and diagnosis of early-stage diabetic kidney disease.

## Figures and Tables

**Table 1:** Comparisons of general characteristics. Data are expressed as means ± SD or median (interquartile range) or number of cases (percent), unless otherwise indicated. 24 h UAE, 24 h urinary albumin excretion; ACEI/ARB, angiotensin-converting enzyme inhibitor/angiotensin II receptor blocker; ALT, alanine aminotransferase; AST, aspartate aminotransferase; β2-MG,β2-microglobulin; BMI, body mass index; DBP, diastolic blood pressure; DM, diabetes mellitus; eGFR, estimated glomerular filtration rate; GGT, γ-glutamyltransferase; HbA1c, haemoglobin A1c; HDL, high-density lipoprotein; IgG, immunoglobulin G; LDL, low-density lipoprotein; RBP, retinol-binding protein; SBP, systolic blood pressure; SUA, serum uric acid; TC, total cholesterol; TG, triglyceride. Comparisons between urine IgG ≤ 2.45 mg/L and urine IgG > 2.45mg/L group are performed using the Mann–Whitney *U* test for continuous data, and chi-square test for categorical data.

Baseline 24 h UAE *<* 30 mg/24 h (*n* = 733)				Baseline 24 h UAE *≥* 30 mg/24 h (*n* = 302)		
Characteristics	Baseline Urine IgG ≤ 2.45 mg/L	Baseline Urine IgG > 2.45 mg/L	*p* Value	Baseline Urine IgG ≤ 2.45 mg/L	Baseline Urine IgG > 2.45 mg/L	*p* Value
**Male (** ***n*** **, %)**	278 (60.4%)	163 (59.7%)	0.846	20 (46.5%)	170 (65.6%)	0.016
**Age**	55.91 ± 10.48	56.81 ± 10.07	0.257	56.24 ± 12.04	54.34 ± 12.19	0.344
**BMI (kg/m^2^)**	26.27 ± 3.65	27.02 ± 3.84	0.009	27.77 ± 4.23	27.85 ± 3.84	0.908
**DM duration (years)**	10.95 ± 6.76	10.71 ± 6.89	0.656	11.92 ± 6.93	10.49 ± 7.18	0.224
**SBP (mmHg)**	130.90 ± 14.83	133.78 ± 16.41	0.015	134.49 ± 15.42	137.63 ± 16.77	0.252
**DBP (mmHg)**	79.29 ± 9.82	80.61 ± 9.65	0.077	82.11 ± 10.40	82.43 ± 9.77	0.844
**HbA1c (%)**	8.41 ± 1.83	8.57 ± 1.69	0.259	8.93 ± 1.91	8.83 ± 1.79	0.716
**eGFR** **(mL/min/1.73 m^2^)**	101.02 ± 13.45	99.33 ± 13.38	0.098	105.61 ± 16.22	99.23 ± 17.77	0.028
**ALT (IU/L)**	23.76 ± 14.83	23.84 ± 13.43	0.945	23.27 ± 17.11	24.68 ± 16.06	0.600
**AST (IU/L)**	20.37 ± 8.85	20.31 ± 9.59	0.930	19.37 ± 9.73	20.23 ± 9.46	0.583
**ALT/AST**	1.14 ± 0.40	1.17 ± 0.36	0.44	1.16 ± 0.33	1.20 ± 0.43	0.547
**GGT (U/L)**	22.50 (15.20–37.00)	26.50 (17.65–37.75)	0.021	26.00 (15.20–38.90)	25.95 (18.50–40.23)	0.617
**SUA (μmol/L)**	308.30 ± 85.41	311.71 ± 85.39	0.601	328.71 ± 94.00	335.42 ± 84.19	0.635
**TC (mmol/L)**	4.87 ± 1.00	5.03 ± 1.29	0.064	5.35 ± 1.44	5.21 ± 1.32	0.522
**TG (mmol/L)**	1.41 (1.03–2.16)	1.54 (1.12–2.33)	0.029	1.95 (1.37–3.77)	1.93 (1.25–3.34)	0.679
**HDL (mmol/L)**	1.25 ± 0.30	1.20 ± 0.26	0.048	1.18 ± 0.26	1.18 ± 0.27	0.978
**LDL (mmol/L)**	3.06 ± 0.81	3.09 ± 1.05	0.062	3.34 ± 1.18	3.25 ± 1.00	0.597
**Smoking (** ***n*** **, %)**	9(2.0%)	3(1.1%)	0.560	0(0.0%)	6(2.3%)	0.599
**Retinopathy (** ***n*** **, %)**	185 (40.2%)	102 (37.4%)	0.444	15 (34.9%)	115 (44.4%)	0.243
**ACEI/ARB use (** ***n*** **, %)**	12(2.6%)	10(3.7%)	0.419	4(9.3%)	12(4.6%)	0.369
**Statin use (** ***n*** **, %)**	4(0.9%)	1(0.4%)	0.656	0(0.0%)	3(1.2%)	1.000
**Urine IgG (mg/L)**	0.68 (0.21–1.36)	5.10 (3.44–7.98)	<0.001	1.14 (0.58–1.78)	11.98 (6.46–27.64)	<0.001
**Urine RBP (mg/L)**	0.15 (0.06–0.32)	0.37 (0.18–0.68)	<0.001	0.18 (0.11–0.32)	0.77 (0.29–1.61)	<0.001
**Urine β2-MG (mg/L)**	0.10 (0.06–0.18)	0.16 (0.09–0.38)	<0.001	0.11 (0.07–0.26)	0.16 (0.08–0.46)	0.07
**24 h UAE (mg/24 h)**	11.05 (5.29–15.00)	14.30 (10.15–20.35)	<0.001	55.38 (41.14–74.24)	87.70 (52.65–149.20)	<0.001
**Follow-up time (years)**	4.16 ± 0.88	4.23 ± 0.85	0.303	4.45 ± 0.92	4.28 ± 0.94	0.288

**Table 2:** Rates of onset and progression of DKD. Data are expressed by the number of cases (percent) used by the chi-square test. 24 h UAE, 24-h urinary albumin excretion; DKD, diabetic kidney disease; IgG, immunoglobulin G. Among patients with baseline 24 h UAE < 30 mg/24 h, those with eGFR ≥ 60 mL/min/1.73 m^2^ and 24 h UAE < 30 mg/24 h at the end of follow-up were categorised as ‘no onset’, those with eGFR < 60 mL/min/1.73 m^2^ and 24 h UAE < 30 mg/24 h were categorised as ‘onset1’, those with eGFR ≥ 60 mL/min/1.73 m^2^ and 24 h UAE ≥ 30 mg/24 h were categorised as ‘onset2’, and those with eGFR < 60 mL/min/1.73 m^2^ and 24 h UAE ≥ 30 mg/24 h were categorised as ‘onset3’. Among patients with baseline 24 h UAE between 30 and 300 mg/24 h, those with eGFR ≥ 60 mL/min/1.73 m^2^ and 24 h UAE < 300 mg/24 h at the end of follow-up were categorised as ‘nonprogress’, those with eGFR < 60 mL/min/1.73 m^2^ and 24 h UAE < 300 mg/24 h were categorised as ‘progress1’, those with eGFR ≥ 60 mL/min/1.73 m^2^ and 24 h UAE ≥ 300 mg/24 h were categorised as ‘progress2’, and those with eGFR < 60 mL/min/1.73 m^2^ and 24 h UAE ≥ 300 mg/24 h were categorised as ‘progress3’.

	Baseline 24 h UAE < 30 mg/24 h (*n* = 733)					Baseline 24 h UAE ≥ 30 mg/24 h (*n* = 302)			
Outcomes	Baseline Urine IgG ≤ 2.45 mg/L	Baseline Urine IgG > 2.45 mg/L	χ2	*p*-Value	Outcomes	Baseline Urine IgG ≤ 2.45 mg/L	Baseline Urine IgG > 2.45 mg/L	χ2	*p*-Value
**No Onset** (*n* = 595)	402 (67.6%)	193 (32.4%)	39.565	<0.001	**Nonprogress** (*n* = 207)	40 (19.3%)	167 (80.7%)	14.104	0.003
**Onset1** (*n* = 14)	10 (71.4%)	4 (28.6%)			**Progress1** (*n* = 15)	1 (6.7%)	14 (93.3%)		
**Onset2** (*n* = 109)	45 (41.3%)	64 (58.7%)			**Progress2** (*n* = 58)	2 (3.4%)	56 (96.6%)		
**Onset3** (*n* = 15)	3 (20.0%)	12 (80.0%)			**Progress3** (*n* = 22)	0 (0.0%)	22 (100.0%)		

**Table 3:** The relationship between urine IgG > 2.45 mg/L and the onset and progression of DKD. ^a^ Adjusted for age, ALT, AST, ALT/AST, BMI, DBP, DM duration, Follow-up time, GGT, HbA1c, HDL, LDL, SBP, SUA, SUrea, TC, TG, uβ2-MG, and uRBP. ^b^ Compared to no onset (*n* = 595). ^c^ Compared to nonprogress (*n* = 207). 24 h UAE, 24 h urinary albumin excretion; ALT, alanine aminotransferase; AST, aspartate aminotransferase; BMI, body mass index; DBP, diastolic blood pressure; GGT, Glutamyl transpeptidase; HbA1c, haemoglobin A1c; HDL, high-density lipoprotein; IgG, immunoglobulin G; LDL, low-density lipoprotein; SBP, systolic blood pressure; SUA, serum uric acid; SUrea, serum urea; TC, total cholesterol; TG, triglyceride uβ2-MG, urine β2-microglobulin; uRBP, urine retinol-binding protein. —, in the population with 24-h UAE ≥ 30 mg/24 h, a logistic regression analysis could not be performed because there were no cases of progress3B in the urine IgG ≤ 2.45 mg/L group. Among patients with baseline 24 h UAE < 30 mg/24 h, those with eGFR ≥ 60 mL/min/1.73 m^2^ and 24 h UAE < 30 mg/24 h at the end of follow-up were categorised as ‘no onset’, those with eGFR < 60 mL/min/1.73 m^2^ and 24 h UAE < 30 mg/24 h were categorised as ‘onset1’, those with eGFR ≥ 60 mL/min/1.73 m^2^ and 24 h UAE ≥ 30 mg/24 h were categorised as ‘onset2’, and those with eGFR < 60 mL/min/1.73 m^2^ and 24 h UAE ≥ 30 mg/24 h were categorised as ‘onset3’. Among patients with baseline 24 h UAE between 30 and 300 mg/24 h, those with eGFR ≥ 60 mL/min/1.73 m^2^ and 24 h UAE < 300 mg/24 h at the end of follow-up were categorised as ‘nonprogress’, those with eGFR < 60 mL/min/1.73 m^2^ and 24 h UAE < 300 mg/24 h were categorised as ‘progress1’, those with eGFR ≥ 60 mL/min/1.73 m^2^ and 24 h UAE ≥ 300 mg/24 h were categorised as ‘progress2’, and those with eGFR < 60 mL/min/1.73 m^2^ and 24 h UAE ≥ 300 mg/24 h were categorised as ‘progress3’.

	Baseline 24 h UAE < 30 mg/24 h (*n* = 733)							Baseline 24 h UAE ≥ 30 mg/24 h (*n* = 302)			
		Univariate Analysis		Multivariate Analysis				Univariate Analysis		Multivariate Analysis	
Outcomes	Baseline Urine IgG	OR (95% CI)	*p*-Value	OR (95% CI)	*p*-Value ^a^	Outcomes	Baseline Urine IgG	OR (95% CI)	*p*-Value	OR (95% CI)	*p*-Value ^a^
**Onset-1 ^b^** **(*n* = 14)**	**Urine IgG > 2.45 mg/L vs.** **Urine IgG ≤ 2.45 mg/L**	0.833 (0.258–2.688)	0.760	0.609 (0.121–3.067)	0.548	**Progress1 ^c^** **(*n* = 15)**	**Urine IgG > 2.45 mg/L vs.** **Urine IgG ≤ 2.45 mg/L**	3.115 (0.396–24.390)	0.281	3.115 (0.180–52.631)	0.434
**Onset-2 ^b^** **(*n* = 109)**	**Urine IgG > 2.45 mg/L vs.** **Urine IgG ≤ 2.45 mg/L**	2.959 (1.949–4.505)	<0.001	2.617 (1.623–4.219)	<0.001	**Progress2 ^c^** **(*n* = 58)**	**Urine IgG > 2.45 mg/L vs.** **Urine IgG ≤ 2.45 mg/L**	6.711 (1.570–28.571)	0.010	7.353 (1.475–37.037)	0.015
**Onset-3 ^b^** **(*n* = 15)**	**Urine IgG > 2.45 mg/L vs.** **Urine IgG ≤ 2.45 mg/L**	8.333 (2.326–30.303)	<0.001	14.706 (2.188–100.000)	0.006	**Progress3 ^c^** **(*n* = 22)**	**Urine IgG > 2.45 mg/L vs.** **Urine IgG ≤ 2.45 mg/L**	—		—	

## Data Availability

The datasets are included within the article and Supplementary Materials.

## Supplementary Materials

The following supporting information can be downloaded at: https://www.mdpi.com/article/10.3390/jpm13030452/s1, Table S1: The relationship between urine IgG and the onset and progression of DKD. aAdjusted for age, ALT, AST, ALT/AST, BMI, DBP, DM duration, Follow-up time, GGT, HbA1c, HDL, LDL, SBP, SUA, SUrea, TC, TG, uβ2-MG, and uRBP. 24 h UAE, 24 h urinary albumin excretion; ALT, alanine aminotransferase; AST, aspartate aminotransferase; BMI, body mass index; DBP, diastolic blood pressure; GGT, Glutamyl transpeptidase; HbA1c, haemoglobin A1c; HDL, high-density lipopro-tein; IgG, immunoglobulin G; LDL, low-density lipoprotein; SBP, systolic blood pressure; SUA, serum uric acid; SUrea, serum urea; TC, total cho-lesterol; TG, triglyceride uβ2-MG, urine β2-microglobulin; uRBP, urine retinol-binding protein. Among patients with baseline 24 h UAE < 30 mg/24 h, those with eGFR ≥ 60 mL/min/1.73 m^2^ and 24 h UAE < 30 mg/24 h at the end of follow-up were categorised as ‘no onset’; those with eGFR < 60 mL/min/1.73 m^2^ and 24 h UAE < 30 mg/24 h were categorised as ‘onset1’; those with eGFR ≥ 60 mL/min/1.73 m^2^ and 24 h UAE ≥ 30 mg/24 h were categorised as ‘onset2’; and those with eGFR < 60 mL/min/1.73 m^2^ and 24 h UAE ≥ 30 mg/24 h were categorised as ‘onset3’. Among patients with baseline 24 h UAE between 30 and 300 mg/24 h, those with eGFR ≥ 60 mL/min/1.73 m^2^ and 24 h UAE < 300 mg/24 h at the end of follow-up were catego-rised as ‘nonprogress’; those with eGFR < 60 mL/min/1.73 m^2^ and 24 h UAE < 300 mg/24 h were categorised as ‘progress1’; those with eGFR ≥ 60 mL/min/1.73 m^2^ and 24 h UAE ≥ 300 mg/24 h were categorised as ‘progress2’; and those with eGFR < 60 mL/min/1.73 m^2^ and 24 h UAE ≥ 300 mg/24 h were categorised as ‘progress3’.
