# Supplementary material for: Urine Immunoglobin G Greater Than 2.45 mg/L Has a Correlation with the Onset and Progression of Diabetic Kidney Disease: A Retrospective Cohort Study
Source: J Pers Med. 2023 Feb 28;13(3):452. doi: 10.3390/jpm13030452 (PMC10056169; doi:10.3390/jpm13030452)
Supplement: Supplementary file 1 [file jpm-13-00452-s001.zip › jpm-2246472-supplementary.pdf]

Table S1. The relationship between urine IgG and the onset and progression of DKD. [a]Adjusted for age, ALT,AST,ALT/AST,BMI, DBP, DM duration, Follow-up time, GGT, HbA1c, HDL, LDL,SBP, SUA, SUrea, TC, TG, uβ2-MG, and uRBP. 24 h UAE, 24 hour urinary albumin excretion; ALT, alanine aminotransferase; AST, aspartate aminotransferase; BMI, body mass index; DBP, diastolic blood pressure; GGT, Glutamyl transpeptidase ;HbA1c, haemoglobin A1c; HDL, high-density lipoprotein; IgG, immunoglobulin G; LDL, low-density lipoprotein; SBP, systolic blood pressure; SUA, serum uric acid; SUrea, serum urea; TC, total cholesterol; TG, triglyceride uβ2-MG, urine β2-microglobulin ; uRBP, urine retinol-binding protein. Among patients with baseline 24 h UAE < 30 mg/24 h, those with eGFR ≥ 60 mL/min/1.73 m^2 and 24 h UAE <30 mg/24 h at the end of follow-up were categorised as 'no onset'; those with eGFR < 60 mL/min/1.73 m^2 and 24 h UAE < 30 mg/24 h were categorised as 'onset1'; those with eGFR ≥ 60 mL/min/1.73 m^2 and 24 h UAE ≥ 30 mg/24 h were categorised as 'onset2'; and those with eGFR < 60 mL/min/1.73 m^2 and 24 h UAE ≥ 30 mg/24 h were categorised as 'onset3'. Among patients with baseline 24 h UAE between 30 and 300 mg/24 h, those with eGFR ≥ 60 mL/min/1.73 m^2 and 24 h UAE <300 mg/24 h at the end of follow-up were categorised as 'nonprogress'; those with eGFR < 60 mL/min/1.73 m^2 and 24 h UAE < 300 mg/24 h were categorised as 'progress1'; those with eGFR ≥ 60 mL/min/1.73 m^2 and 24 h UAE ≥ 300 mg/24 h were categorised as 'progress2'; and those with eGFR < 60 mL/min/1.73 m^2 and 24 h UAE ≥ 300 mg/24 h were categorised as 'progress3.'

	**Baseline 24 h UAE < 30 mg/24 h (*n* = 733)**						**Baseline 24 h UAE ≥ 30 mg/24 h (*n* = 302)**				
		**Univariate analysis**		**Multivariate analysis**				**Univariate analysis**		**Multivariate analysis**	
**Outc-omes**		**OR (95% CI)**	***p* value**	**OR (95% CI)**	***p* value [a]**	**Outc-omes**		**OR (95% CI)**	***p* value**	**OR (95% CI)**	***p* value [a]**
**Onset- 1 (*n* = 14)**	**BaselineU-rine IgG**	0.996 (0.916–1.084)	0.929	1.013 (0.918–1.118)	0.794	**Progr-ess1 (*n* = 15)**	**BaselineU-rine IgG**	1.010 (0.998 –1.021)	0.104	1.025 (1.002- –1.049)	0.031
**Onset- 2 (*n* = 109)**	**BaselineU-rine IgG**	1.048 (1.025–1.072)	< 0.001	1.039 (1.018–1.061)	< 0.001	**Progr-ess2 (*n* = 58)**	**BaselineU-rine IgG**	1.029 (1.015 –1.044)	< 0.001	1.031 (1.014 –1.048)	< 0.001
**Onset -3 (*n*=15)**	**BaselineU-rine IgG**	1.055 (1.031–1.079)	< 0.001	1.051 (1.028–1.074)	< 0.001	**Progr-ess3 (*n* = 22)**	**BaselineU-rine IgG**	1.032 (1.017 –1.048)	< 0.001	1.022 (1.008 –1.037	0.002
